# Econophysics and the Landauer Principle: A Redefinition of Economic Temperature

**DOI:** 10.3390/e28040376

**Published:** 2026-03-26

**Authors:** Edward Bormashenko, Igor Shendrik

**Affiliations:** 1 Chemical Engineering Department, Ariel University, Ariel 407000, Israel; 2 Department of Dermatology, The University of Oklahoma College of Medicine, Oklahoma City, OK 73104, USA

**Keywords:** Landauer principle, entropy, econophysics, economic temperature, high-frequency trading, Carathéodory principle, economic Szilard engine, Curzon–Ahlborn economic engine

## Abstract

A redefinition of key notions in econophysics based on the Landauer principle is proposed. Marginal economic temperature is defined via the economic Landauer principle and is proportional to the minimal monetary cost associated with erasing or transmitting one bit of information in a given economic system. The actual economic temperature is defined as the effective monetary cost per irreversibly processed bit in a given economic system. The introduced definitions are particularly relevant for high-frequency trading environments. The Clausius formulation of the Second Law is reformulated for economic systems as the statement that money cannot spontaneously flow from a colder economic subsystem to a hotter one. Economic analogues of Carnot and Szilard engines are analyzed. The Carathéodory formulation of the Second Law is reformulated as follows: in every neighborhood of any equilibrium economic state there exist states that cannot be reached by processes that do not expend money or information. An optimal-power Curzon–Ahlborn economic engine is discussed. Illustrative examples of the introduced economic temperature are provided. Finally, a redefinition of economic entropy based on information processing is proposed. First and Third Laws of economic thermodynamics are proposed.

## 1. Introduction

Thermodynamics, i.e., the science devoted to the study of thermal processes, is a relatively young but extremely important field of physics. Our paper is devoted to the extension of main ideas and notions of thermodynamics to econophysics, which is an interdisciplinary field that applies statistical-physics/thermodynamics methods to economic systems in order to identify universal, emergent, and scaling properties of collective economic dynamics [1,2,3,4]. Albert Einstein stated that thermodynamics “is the only physical theory of universal content, which, I am convinced, within the framework of applicability of its basic concepts will never be overthrown” [5]. Rigorously speaking, Einstein is saying: as long as concepts like energy, entropy, temperature, and equilibrium make sense, thermodynamics will not be overthrown. This is crucial. Actually, thermodynamics provide a scientifically robust framework whose validity is established through its stability and consistency across a wide range of macroscopic systems. At the same time, temperature, which is one of the basic notions of thermodynamics, is not defined with the same degree of rigor as the main notions of classical mechanics. Let us briefly survey the definitions of the temperature: (i) the microscopic understanding of temperature is that the temperature of a substance is related to the average kinetic energy of the particles of that substance [6,7,8,9,10,11]; (ii) the macroscopic definition of the temperature emerges from the concept of entropy, and the temperature is defined with the equation:

(1) $$\frac{1}{T}={\left(\frac{\partial S}{\partial E}\right)}_{N},$$

where *E* and *S* are the energy and entropy of the system correspondingly and *N* is the number of particles constituting the given thermodynamic system [11]. It follows from Equation (1), that the macroscopic definition of temperature is not related to the averaging of the kinetic motion of particles constituting the system, and may be introduced for systems containing an arbitrary number of particles [12]. The connection between these definitions is not entirely straightforward. Obviously, the microscopic definition of temperature forbids negative absolute temperatures, whereas the macroscopic definition, supplied by Equation (1), permits them [12]. The problem of the relativistic transformation for the temperature remains open and highly debatable [12,13,14,15]. The review of the problems related to the rigorous definition of temperature was published [12]. There exists one more possibility to define the temperature. This is the possibility based on the Landauer principle [16,17,18,19,20,21,22,23,24,25,26,27,28]. Aphoristically stated, the Landauer principle says, that “information is physical”. In other words, storage/erasure of information is a physical process requiring energy [16,17,18,19,20,21,22,23,24,25,26,27,28]. In its strict sense the Landauer principle in its simplest meaning states that the erasure of one bit of information in a given system requires a minimum energy cost equal to $k_{B}$*Tln*2, where *T* is the temperature at which erasure occurs and $k_{B}$ is the Boltzmann constant [16,17,18,19,20,21,22,23,24,25,26,27,28]. It should be emphasized that the Landauer principle establishes the energetic cost of information erasure, and does not predict the minimal cost of its storage. This information storage/erasure asymmetry is very important [16,17,18,19,20,21,22,23,24,25,26,27,28]. Rolf Landauer also applied the suggested principle to the transmission of information and reshaped it as follows: an amount of energy equal to $k_{B}$*Tln*2 (where $k_{B}$*T* is the thermal noise per unit bandwidth) is needed to transmit a bit of information, and more if quantized channels are used with photon energies *hν* > $k_{B}$*T* [18]. We demonstrate that the Landauer principle enables redefinition of “economic temperature” and reshaping the laws of econophysics.

The aim of this paper is to formulate a physically grounded notion of economic temperature based on the Landauer principle and to investigate how the principal laws of thermodynamics can be reinterpreted for economic systems characterized by explicit informational irreversibility. The object of the study is therefore not the economy in its full behavioral, institutional, or macroeconomic complexity, but rather a class of information-intensive economic systems in which decisions are realized through storage, transmission, processing, and erasure of information with measurable costs. High-frequency trading, electronic markets, and ledger-based financial infrastructures are employed as primary examples because they provide the clearest realization of these conditions.

In the present work, we define an economy as a physical information-processing system composed of interacting agents that allocate resources through decisions based on information. Each agent acquires, stores, processes, and transmits information regarding prices, risks, and opportunities, and economic transactions represent the outcomes of these information-processing operations. Since information processing is physically implemented (for example, in human cognition, digital computation, communication networks, and financial infrastructure), it necessarily involves irreversible operations such as information erasure and updating. According to the Landauer principle, such operations carry a minimal thermodynamic cost. Consequently, economic activity may be viewed as a network of information flows whose physical implementation generates dissipation, enabling the introduction of a Landauer-based economic temperature.

The paper is built as follows: (i) we define the notion of the economic temperature via the economic Landauer principle; (ii) Clausius interpretation of the Second Law of Economic Thermodynamic is discussed; (iii) Carathéodory interpretation of the Second Law of Economic Thermodynamics is suggested; (iv) Carnot, Szilárd and Curzon–Ahlborn economic engines are introduced and analyzed; (v) economic entropy is re-defined; (vi) limitations of the introduced economic temperature are discussed; (vii) trends of future investigations are envisaged; (vi) first and third laws of economic thermodynamics are suggested.

## 2. Results

### 2.1. Redefinition of the Economic Temperature

There is no unified approach to the definition of temperature in economic systems. Temperature is introduced in economics as: (i) measure of inverse rationality/the degree of randomness (bounded rationality) in agents’ decisions [29], (ii) price volatility/market agitation [30], (iii) measure of the scale of wealth fluctuations [31], (iv) inverse market efficiency [32], (v) definition exploiting the energy/entropy of economic systems. In this case, the economic temperature is defined as:

(2) $$T=-{\left(\frac{\partial U}{\partial S}\right)}_{X},$$

where *U* is the utility/energy, *S* is the entropy and *X* defines state variables [33]. This definition resembles, but does not coincide with, that supplied by Equation (1). In the econophysical analogy proposed here, *U* represents the economic potential of the system, which may be interpreted as utility or extractable economic value. The analogy is therefore structural rather than literal: utility plays the role of a thermodynamic potential measured in monetary units, whereas entropy *S* characterizes informational uncertainty in economic decision processes. Consequently, informational entropy reduction in decision-making entails physical energy dissipation and corresponding economic costs, which motivates the introduction of an economic temperature measured in monetary units per processed bit. The redefinition of economic entropy will be supplied below in Section 2.7.

We define the “economic temperature” with the Landauer principle, based on the thermodynamics of information. Consider a given economic system. Erasure of a single bit of information of this system according to the Landauer principle requires the minimal energy given by $E=ln2k_{B}T$. We re-write this equation as follows:

(3) $$\hat{E}=T^{*}ln2,$$

where $\hat{E}$ is the cost of electrical power necessary for erasure of one bit of information, $\left[\hat{E}\right]=\frac{\$}{\mathrm{bit}}$ and $T^{*}$ is the marginal economic temperature, $\left[T^{*}\right]=\frac{\$}{\mathrm{bit}}$. The quantity $T^{*}$ represents the marginal monetary cost associated with irreversible information processing in the given economic system.

It is convenient to keep the multiplier ln2 in Equation (3), resembling the Landauer-like definition of temperature. Obviously, the marginal economic temperature is given by: $T^{*}=\frac{\hat{E}}{ln2}.$ Equation (3) provides the unique lower bound that follows from a fundamental constraint—logical irreversibility—rather than from contingent implementation losses. Landauer’s principle states that whenever an operation many-to-one maps memory states (reset/overwrite/decision finalization), the information-bearing degrees of freedom lose at least ln2 of Shannon entropy per erased bit, and the environment must gain at least the same entropy; hence a minimal dissipated energy $E=ln2k_{B}T$. Our economic definition (see Equation (3)) simply re-expresses this irreducible cost in monetary units, $\hat{E}=ln2T^{*}$. Any other “loss term” (fees, spreads, latency penalties, hardware inefficiency) is system-specific and can vanish in an idealization, but Landauer cannot for a cyclic operation that requires memory reset. Therefore Equation (3) plays the same role as in thermodynamics: it provides a model-independent baseline for dissipation per economically relevant bit; real markets sit above it, so empirically $\hat{E}$ is an effective (measurable) marginal cost per bit, but the fundamental reason it must be nonzero is precisely Landauer-principle-grounded.

Let us establish interrelation between the marginal economic temperature $T^{*}$ and standard Kelvin temperature *T*. The cost of electrical power necessary for erasure of one bit of information may be expressed with the use of the Landauer principle as follows:

(4) $$\hat{E}=kk_{B}P_{J}Tln2,$$

where $P_{J}$ is the marginal electricity cost per Joule, *k* is the dimensionless coefficient, which depends on a given economic system, k=1 for the economic system operating at the physical Landauer limit and $k_{B}P_{J}Tln2$ is the cost of erasing of 1 bit for the ideal information processing systems working at the Landauer limit. For any real computing system k>1. According to US Energy Information Administration (EIA) data, the industrial price is $P_{J}^{ind}=0.084\frac{\$}{\mathrm{kWth}}=2.34\times 10^{-8}\frac{\$}{J}$ and the commercial price is $P_{J}^{com}=0.1319\frac{\$}{\mathrm{kWth}}=3.67\times 10^{-8}\frac{\$}{J}$. Comparing Equations (3) and (4) eventually yields:

(5) $$T^{*}=P_{J}kk_{B}T.$$

It follows from Equation (5) that at the fixed temperature *T* the marginal economic temperature $T^{*}$ of the given system (k=const) is completely defined by the electricity price. Let us calculate $T^{*}$ for the industrial and commercial electricity prices for T=300 K, k=1, corresponding to the Landauer limit. We calculate with Equation (5): $T_{ind}^{*}=9.69\times 10^{-29}\frac{\$}{\mathrm{bit}}$ for the industrial price and $T_{com}^{*}=2.52\times 10^{-28}\frac{\$}{\mathrm{bit}}$ for the commercial price. Actually, for real computing systems k≫1, as demonstrated in Appendix A. Thus, real economic systems operate far from the Landauer limit. Distinguishing between marginal and actual economic temperature is useful. The actual economic temperature is, in turn, given by: $T_{act}^{*}=\frac{{\hat{E}}_{act}}{ln2}$, where ${\hat{E}}_{act}$ is an actual minimal cost of erasure on 1 bit of information, in a given economic system, which may be much larger than the marginal cost, defined by the cost of electrical power necessary for the erasure of 1 bit of information, i.e., $T_{act}^{*}\gg T^{*}$ often takes place.

The natural questions are: when and why should the definition work? The definition of economic temperature (marginal and actual) $T^{*}=\frac{\hat{E}}{ln2}$ is meaningful for economic systems that satisfy the following conditions:

(i) Information is explicitly stored, processed, erased, or transmitted;
(ii) Information-processing events have a measurable economic cost;
(iii) The system operates close to an efficiency limit, so minimal costs matter.

The Landauer-based definition of economic temperature will apply to economic systems that operate as information-processing structures, in which decisions, signals, or records are logically irreversible and incur a minimal, system-dependent economic cost. For example, it is well-expected to work for high-frequency trading (HFT), which is an automated trading strategy characterized by ultra-low latency, high-order submission and cancellation rates, very short holding periods, and profits derived from small price differentials rather than long-term asset valuation [34]. HFT is often defined as a strategy that responds to market events in the millisecond environment. It is important that there is typically no human intervention per trade within HFT. In HFT, a single completed trade—or equivalently, a single finalized decision—can be modeled as one logical operation, corresponding to the erasure of at least one bit of information:

(6) trade↔logical decision↔bit erasure.

Typical profit per trade is tiny on HFT: fractions of a cent, and trading decisions are literally bit operations. In 2016, HFT on average initiated 10–40% of trading volume in equities, and 10–15% of volume in foreign exchange and commodities [35]. Actual economic temperature $T_{act}^{*}=\frac{{\hat{E}}_{act}}{ln2}$ is interpreted as follows: high $T_{act}^{*}$ corresponds to noisy, fast, wasteful markets, low $T_{act}^{*}$ corresponds to slow, efficient, information-preserving markets. Thus, bitcoin-like systems correspond to extremely high $T_{act}^{*},$ as demonstrated in Appendix B. ${\hat{E}}_{act}$ is in turn understood as the minimal transaction or validation cost (see Appendix B). The introduced economic temperature is relevant for HFT due to the following reasoning: (i) information processing has a real monetary and energy cost, (ii) in HFT, erasing a bit too slowly or too expensively means losing money. Thus, we conclude that: the definition of economic temperature supplied by Equation (3) does not rely on agent rationality, equilibrium assumptions, or utility maximization. Instead, it rests on a structural property common to both thermodynamic and economic systems: decisions destroy information, and information destruction has an irreducible cost. Hence, HFT systems may be viewed as economic Landauer engines, converting information erasure into monetary dissipation. This establishes a direct, physically grounded definition of economic temperature and provides a microscopic foundation for extending thermodynamic reasoning to econophysics. Why is the introduced economic temperature expected to work? It works due to the fact that decisions erase alternatives, giving rise to logical irreversibility.

### 2.2. Re-Interpretation of the Second Law of Thermodynamics

One of the possible formulations of the second law of thermodynamics belongs to Rudolf Clausius and is as follows: heat cannot spontaneously flow from a colder body to a hotter body. Let us re-formulate the Second Law of thermodynamics for economic systems for which the economic temperature may be introduced by Equation (3). This formulation is shaped as follows: energy/money cannot spontaneously flow from a colder economic system to a hotter economic system [2]. Let us exemplify this interpretation:

(i) High-frequency trading vs. long-term investors. Consider two economic subsystems, namely: Hot system: high-frequency trading (HFT) firms operating at high actual economic temperature $T_{HFT}^{*}$, characterized by ultra-low latency, massive order cancellation, and frequent logical erasure events, and cold system: long-term institutional or retail investors operating at a much lower actual economic temperature $T_{LT}^{*}$, characterized by infrequent decisions and low information-processing dissipation. Empirically, money does not spontaneously flow from long-term investors to HFT firms in the absence of informational or structural asymmetry. Instead, HFT extracts value from hot microstructure inefficiencies, i.e., latency gaps, order-book imbalances, and transient arbitrage opportunities, i.e., generated within similarly hot or hotter market segments. A cold investor placing a buy-and-hold order does not continuously transfer money to HFT traders; the value transfer occurs only when the cold system is forced into a hotter regime, e.g., by reacting to short-term price fluctuations, stop-loss triggers, or liquidity shocks. In thermodynamic terms, the cold system must be locally heated before energy (money) can flow. It seems that this conclusion contradicts the Clausius interpretation of the Second Law of Thermodynamics. The apparent contradiction with the Clausius formulation disappears once one recognizes that, both in physics and in economic thermodynamics, energy (money) flows only along existing coupling channels; in economic systems, such coupling is created when a cold agent is locally driven into a high-frequency information-processing regime.
(ii) Latency arbitrage in HFT. Latency arbitrage in HFT is a trading strategy that exploits minimal time differences in market data and trade execution across different trading venues. This practice involves detecting price discrepancies between markets and acting on them before they naturally resolve, typically operating at microsecond or nanosecond timescales. Latency arbitrage in HFT may be interpreted as an economic analogue of heat flow from hot to cold. In this case, a hot subsystem rapidly processes and erases information about price changes across exchanges; a colder subsystem, in turn, updates prices more slowly. Profits arise when HFT firms exploit temporary price discrepancies between exchanges operating at different effective temperatures. Crucially, the money flows from the hotter, noisier information-processing layer to the colder, more stable layer only after dissipation occurs in the hot system (computational cost, infrastructure cost, fees). No spontaneous reverse flow is observed: a slower, colder exchange cannot systematically extract value from a faster, hotter one without increasing its own information-processing rate and cost—i.e., without increasing its economic temperature.
(iii) Cryptocurrency markets vs. traditional financial systems. Cryptocurrency markets provide an example of extremely high economic temperature $T_{act}^{*}$ (see Appendix B). Transaction validation, mining, and consensus mechanisms involve enormous information erasure costs and energy dissipation. Traditional banking systems, in contrast, operate at much lower economic temperatures. Any finite-rate transfer across any real economic channel produces dissipation, just as in physical thermodynamics. On-chain cryptocurrency settlement constitutes an exceptionally “hot” channel: the marginal cost of committing information, measured in $\frac{\$}{\mathrm{bit}}$ to the ledger (derived from fees per vB), is typically orders of magnitude larger than that of many conventional payment/communication rails (see Appendix A, Appendix B and Appendix C). Therefore, reallocating capital into on-chain activity generally implies operating through a higher-$T_{act}^{*}$ coupling, which—relative to colder rails—entails larger irreversible costs and, frequently, increased short-horizon exposure to microstructure and price variability. Analogously to heat transport, where both temperature difference and thermal conductance determine flux and dissipation, economic value transfer depends on both $T_{act}^{*}$ gradients and coupling strength (fees, bandwidth, latency sensitivity, access constraints). In effect, funds must be “heated” (subjected to higher $T_{act}^{*}),$ before they can circulate in the hotter system.
(iv) Trading fees and market frictions as entropy production. Transaction fees, bid–ask spreads, and slippage play the role of entropy production in economic systems [36,37] (the economic entropy will be discussed in detail in Section 2.7). They ensure that a closed economic cycle cannot yield net profit without dissipation. A trader attempting to extract value from a high-frequency, high-temperature market must necessarily incur informational and transactional costs associated with information acquisition, transmission, and decision execution. In the Landauer-based framework introduced here, these costs correspond to the minimal irreversible dissipation per economically relevant bit, quantified by the economic temperature $T_{act}^{*}$. The claim is therefore not merely that real economic processes are irreversible, but that no costless (economic-adiabatic) path exists for systematic profit extraction from a hot market, which is the economic analogue of the thermodynamic statement that work cannot be extracted from a single reservoir without dissipation.

Finally, the economic Clausius principle (or Clausius interpretation of the Second Economic Law of Thermodynamics) may be summarized as follows: economic value flows from hot to cold only via dissipation, and never spontaneously from cold to hot.

This principle does not depend on agent rationality or equilibrium assumptions. Instead, it emerges from the logical irreversibility of economic decisions and the Landauer cost associated with information erasure. In this sense, the Second Law of Thermodynamics survives in economics in an informationally grounded form, with economic temperature replacing physical temperature. Just as heat flows from hot to cold unless work is performed, money flows from high-temperature economic subsystems to lower-temperature ones only through irreversible information processing and dissipation.

Now we address the Constantin Carathéodory interpretation of the Second Law of Economic Thermodynamics, which is formulated as follows: In every neighborhood of any equilibrium state, there exist states that cannot be reached by adiabatic processes. “Adiabatic” means without heat exchange [38,39,40]. The crucial idea is inaccessibility, not entropy or heat flow [38,39,40].

First of all, we have to re-define the adiabatic process in the terms of economic thermodynamics. When economic temperature is defined via the Landauer principle, the natural economic analogue of an adiabatic process is a process that does not spend money or information, i.e., does not incur transaction costs, fees, latency costs, computational effort, or information erasure. We also have to define the “economic state”. A thermodynamic economic state is defined as an equivalence class of microscopic economic configurations characterized by identical values of aggregate resources, information content, economic entropy, number of active degrees of freedom *N* (number of active agents, instruments, strategies, or contracts participating in the dynamics), and institutional constraints.

(7) $$X=\left(W,I,S_{e},N,\Lambda \right),$$

where *W* is—aggregate economic energy (total available economic resources capable of doing economic “work” (capital, liquidity, etc.), *I* is information content (total usable information held by agents and infrastructures), $S_{e}$ is economic entropy, which is a measure of dispersion, uncertainty, or irreversibility in the economic system (wealth distribution spread, order-book disorder, transaction irreversibility, information loss) and Λ denotes institutional and technological constraints (market rules, transaction costs, latency, regulation, clearing mechanisms, and technological limits (including Landauer-type limits)).

The Carathéodory principle is re-shaped as follows: in every neighborhood of any equilibrium economic state, there exist states that cannot be reached by the process, which does not spend money or information. When economic temperature is defined via the Landauer principle, the natural economic analogue of an adiabatic process is a process that does not spend money or information, i.e., does not incur transaction costs, fees, latency costs, computational effort, or information erasure.

Consider the HFT example. Consider an electronic market operating near a local equilibrium state characterized by: (i) a stable mid-price, (ii) a fixed bid–ask spread, (iii) balanced order-book depth, (iv) no net arbitrage opportunities. This equilibrium corresponds to a point in the economic state space. Now consider an arbitrarily small neighborhood of this state—for example, a configuration with a slightly tighter spread, slightly increased liquidity at the best bid, or a slightly improved execution probability. The Carathéodory principle asserts that some of these nearby states are inaccessible without dissipation. Indeed, in HFT one cannot tighten the bid–ask spread, even infinitesimally, without submitting additional orders. Submitting or canceling orders necessarily consumes computational resources, incurs exchange fees, and erases information (decisions overwrite alternatives). Therefore, reaching certain nearby market configurations is impossible without spending money or information. A “free” trajectory through economic state space does not exist, even locally. The equilibrium is surrounded by inaccessible states unless economic work is performed. This is a direct analogue of the classical Carathéodory statement: the neighborhood of equilibrium is not adiabatically connected.

Consider one more example. Consider an equilibrium market state in which all publicly available information is reflected in prices. In an arbitrarily small neighborhood of this equilibrium lie states in which a trader holds a slightly positive arbitrage position. Carathéodory’s economic principle asserts that such nearby profitable states cannot be reached without spending resources. Any attempt to exploit arbitrage requires placing orders, paying fees, absorbing slippage, and processing information. A “zero-cost” arbitrage path is forbidden. The impossibility of costless arbitrage is thus an accessibility restriction, not merely a profit statement.

And, eventually we address one more example, namely: portfolio rebalancing near equilibrium. Consider an investor holding a diversified portfolio at equilibrium relative to a benchmark. In the neighborhood of this state lie portfolios with marginally higher expected return or lower risk. Reaching these states requires: transactions, information gathering, model updates, reallocation decisions. A hypothetical “adiabatic” rebalancing is one that changes the portfolio without trading, information processing, or decision-making. Such a rebalancing is impossible. Thus, even infinitesimal improvements in portfolio configuration are not freely accessible.

Thus, we conclude that equilibrium points in the economic space are surrounded by inaccessible directions unless money or information is expended. Thus, the Carathéodory formulation of the Second Law translates naturally into economics: every equilibrium economic state is surrounded by nearby states that cannot be reached without spending money or information, reflecting the fundamentally non-adiabatic nature of economic transformations.

### 2.3. Economic Carnot Engine

One more classical formulation of the Second Law of Thermodynamics is due to Sadi Carnot and is based on the concept of an ideal heat engine operating between two reservoirs at different temperatures. In classical thermodynamics, a Carnot engine extracts work from the heat flow between a hot reservoir at temperature $T_{H}$ and a cold reservoir at temperature $T_{C}$. The Carnot theorem states that no engine operating between two reservoirs can be more efficient than a reversible Carnot engine, whose efficiency is given by:

(8) $$\eta_{Carnot}=1-\frac{T_{C}}{T_{H}}.$$

This formulation does not rely on entropy production explicitly; instead, it is rooted in the impossibility of extracting unlimited work from a finite temperature difference. We now construct the economic analogue of a Carnot engine using the Landauer-based definition of economic temperature $T^{*}.$ An economic Carnot engine is defined as an idealized information-processing economic system that operates cyclically between two economic reservoirs characterized by different marginal economic temperatures $T_{H}^{*}>T_{C}^{*}$. The hot economic reservoir corresponds to a high-temperature economic subsystem, such as HFT, cryptocurrency mining, or high-volatility speculative markets. These systems are characterized by rapid information erasure, high transaction intensity, and significant monetary dissipation per decision. The cold economic reservoir corresponds to a low-temperature economic subsystem, such as long-term investment strategies, slow institutional capital, or stable banking systems, characterized by infrequent decision-making and low information-processing dissipation. The working substance of the economic Carnot engine is information: signals, orders, strategies, and decisions. Economic work corresponds to net monetary profit, whereas economic heat corresponds to irreversible monetary dissipation associated with information erasure, transaction costs, fees, latency, and computational effort.

Let ${\hat{E}}_{H}$ be the monetary cost associated with information erasure when interacting with the hot reservoir, and ${\hat{E}}_{C}$ be the corresponding cost when interacting with the cold reservoir. According to the Landauer-based definition, the costs are given by Equation (3). The net economic work extracted in one cycle is defined by Equation (9):

(9) $${\hat{W}}^{*}={\hat{E}}_{H}-{\hat{E}}_{C},$$

where ${\hat{E}}_{H}=T_{H}^{*}ln2$ and ${\hat{E}}_{C}=T_{C}^{*}ln2$. The economic $\eta_{ec}$ efficiency of the engine is then defined as (Equation (5) is involved):

(10) $$\eta_{ec}=\frac{{\hat{W}}^{*}}{{\hat{E}}_{H}}=\frac{{\hat{E}}_{H}-{\hat{E}}_{C}}{{\hat{E}}_{H}}=1-\frac{T_{C}^{*}}{T_{H}^{*}}=1-\frac{k_{C}P_{JC}T_{C}}{k_{H}P_{JH}T_{H}},$$

where $P_{JC}$ and $P_{JH}$ are the marginal electricity cost per Joule at the cold and hot economic reservoirs correspondingly, $k_{C}$ and $k_{H}$ are the dimensionless constants depending on the cold and hot economic systems respectively, appearing in Equation (4). In the particular case (and only in this case), when $k_{C}P_{JC}=k_{H}P_{JH}$, we extract the classical Carnot formula:

(11) $$\eta_{ec}=1-\frac{T_{C}}{T_{H}}.$$

This result supplied by Equation (10) is very strong: the mathematical structure of the Carnot bound survives unchanged when physical temperature is replaced by Landauer-defined marginal economic temperature. For the real economic systems, the marginal economic temperatures should be replaced by the actual ones, denoted $T_{actC}^{*}$ and $T_{actH}^{*}$, respectively. Thus, the efficiency is supplied by: $\eta_{ec}=1-\frac{T_{actC}^{*}}{T_{actH}^{*}}.$ Consider HFT as an economic heat engine. HFT systems act as engines that extract profit from price gradients, latency differences, and microstructure inefficiencies. These gradients exist primarily between market segments operating at different economic temperatures. An HFT firm absorbs high-temperature information noise (rapid price fluctuations, order-book changes), performs logical erasure through decision-making, and converts part of this dissipation into monetary profit. The remaining part is lost as fees, infrastructure costs, and energy consumption. The Carnot bound implies that there exists a strict upper limit on the profitability of such strategies, determined by the ratio $\frac{T_{actC}^{*}}{T_{actH}^{*}}.$

Another example is delivered by cryptocurrency mining. Cryptocurrency networks represent extremely hot economic reservoirs, with enormous information-erasure costs per validated block (see Appendix B). Traditional financial systems act as colder reservoirs. Any attempt to extract profit by arbitraging between these systems is subject to a Carnot-type bound: no strategy can convert the high dissipation of the hot system into monetary gain with efficiency exceeding $\eta_{ec}=1-\frac{T_{actC}^{*}}{T_{actH}^{*}}$. The Carnot formulation immediately forbids economic perpetual motion machines of the second kind: strategies that claim to extract profit from information processing without dissipation, or with efficiency exceeding the Carnot-type bound. Any such strategy will require $\eta_{ec}>1-\frac{T_{actC}^{*}}{T_{actH}^{*}}$, which is impossible. Thus, schemes promising risk-free arbitrage without transaction costs, latency costs, or information-processing effort are economic analogues of thermodynamic perpetual motion machines and are forbidden by the Landauer-based Economic Second Law. Therefore, the economic Carnot principle demonstrates that profit extraction is fundamentally constrained by information thermodynamics. Economic systems may be viewed as information engines, and markets as thermal environments, in which monetary profit plays the role of work and transaction costs play the role of waste heat.

The proposed economic Carnot bound applies to ensemble-averaged performance, not necessarily to every individual trade. In fluctuating markets, rare “super-efficient” trades may occur, in which the realized profit temporarily exceeds the average dissipation-based bound because of noise, transient arbitrage, or favorable microstructure fluctuations. These events are the economic analogue of negative entropy-production fluctuations in stochastic thermodynamics. However, they are statistically compensated by opposite fluctuations and therefore do not violate the average economic Second Law, which constrains the mean efficiency and mean dissipation of repeated trading cycles.

To make the Carnot analogy more concrete, consider a numerical HFT example using actual exchange-level cost components. For instance, Nasdaq lists a 10 Gb colocation connection at 11,000 $/month, cabinet space at 165 $ per U per month, and a Nasdaq Depth Non-Display Enterprise License (Direct Access) at 75,000 $/month, while a PSX (Nasdaq PHLX (Philadelphia Stock Exchange) TotalView Non-Display is 18,190 $/month with 1110 $/month direct access [41]. If a strategy processes $10^{9}$ decision-relevant bits per day over 20 trading days, an 8U Nasdaq stack corresponds to $T_{actH}^{*}=4.4\times 10^{-6}\frac{\$}{\mathrm{bit}}$, whereas the PSX stack gives $T_{actC}^{*}=9.7\times 10^{-7}\frac{\$}{\mathrm{bit}}$. This yields an economic Carnot bound $\eta_{Carnot}=1-\frac{T_{ACTC}^{*}}{T_{ACTH}^{*}}=0.78.$ Thus, for a gross informational inflow of 50,000 $/month, the maximal reversible profit is about 39,000 $/month, while about 11,000 $/month must be dissipated. We now state explicitly that this is an order-of-magnitude empirical illustration, not a claim of exact universality. The scale is consistent with the very high message volumes observed in modern equity-market data systems [41].

### 2.4. Alternative Understanding of the Economic Temperature

We do not believe that a unique definition of economic temperature is possible. Therefore, we introduce an alternative definition based on the Landauer principle. We still define marginal economic temperature $T^{*}$ with the Landauer-like limit supplied with Equation (3), namely: $\hat{E}=T^{*}ln2$. However, $\hat{E}$ is now the cost of electrical power necessary for transmission of one bit of information. This is the “information friction” based definition of the economic temperature, exploiting the original idea by Rolf Landauer, demonstrating that there exists an energy limit necessary for the transmission of 1 bit of information. Interrelation between economic and thermodynamic temperature is still given by Equation (5), in which *k* is the dimensionless constant depending on the channel of the information transmission. Again, real economic systems operate at actual economic temperatures far from the Landauer limit.

Consider a high-frequency trading (HFT) firm operating on an electronic financial exchange. The firm’s profit critically depends on the transmission, processing, and erasure of information—order-book updates, price changes, and trading signals—at extremely high rates. Each economically relevant event (e.g., best bid changes by one tick) corresponds to a binary decision at the level of the trading algorithm: (i) act/do not act; (ii) submit order/cancel order; and (iii) route order/suppress order. Thus, the trading process naturally decomposes into a stream of bit-level operations. Information friction and minimal energy cost. Transmission of a single bit from the exchange to the trading engine (and further to the execution venue) is not free. It requires electrical energy for signal generation and amplification, energy for error correction and noise suppression, and irreversible logical operations (bit erasure, overwriting buffers, cache clearing).

Even in the ideal limit, the minimal electrical energy cost per transmitted bit is bounded from below. In the spirit of the Landauer principle, we write this marginal bound as $\hat{E}=T^{*}ln2.$ The actual economic temperature $T_{act}^{*}$ measures the irreducible monetary cost of fast information flow in the market. A high $T_{act}^{*}$ market is one where even a single bit of actionable information is expensive to transmit (high energy prices, regulatory overhead, latency penalties, infrastructure scarcity). A low $T_{act}^{*}$ market corresponds to cheap, abundant, and efficient information transmission. It should be emphasized that $T_{act}^{*}$ is not a behavioral or psychological quantity. It is a technological and infrastructural parameter, fixed by: electricity prices, communication hardware, exchange protocols and latency arbitrage competition.

Crucially, this cost is irreversible. Once the bit is transmitted and processed, the spent electrical energy is dissipated as heat and cannot be recovered. This makes high-frequency trading a natural economic realization of Landauer’s information friction, where profits are extracted from information asymmetries, but information processing necessarily produces entropy in the economic sense (transaction costs, fees, infrastructure depreciation).

Thus, the economic temperature $T_{act}^{*}$ quantifies the minimal irreversible monetary cost per bit of information, providing a physically grounded and operational definition of temperature in economic systems. This interpretation of the economic Landauer principle is directly bridged to the Carathéodory principle (see Section 2.2). Consider two economic states. Although the difference between the two states is arbitrarily small in economic terms, reaching the neighboring state requires the acquisition and processing of at least one bit of information (e.g., detecting a price change and reacting to it). By the Landauer-like bound, transmission of this single bit necessarily costs at least $\hat{E}=T_{act}^{*}ln2.$ Therefore, the transition cannot be performed by an economic adiabatic process.

Accordingly, capital flows are expected to preferentially move from hotter informational channels toward colder ones. A real-world illustration of money flow between subsystems with different Landauer-defined economic temperatures is provided by cross-border remittances and stablecoin payment rails. The global average cost of sending 200 $ is about 6.49% corresponding to a fee of about 12.98 $ [42]. Assuming a minimal payment message of order $B≅2\;k_{B}=1.6\times 10^{4}$ bits of economically relevant information, the monetary dissipation per bit is ${\hat{E}}_{rem}=\frac{12.98\;\$}{1.6\times 10^{4}\;\mathrm{bit}}≅8.1\times 10^{-4}\frac{\$}{\mathrm{bit}}$. Using the Landauer-type definition of economic temperature we calculate $T_{rem}^{*}≅1.17\times 10^{-3}\frac{\$}{\mathrm{bit}}.$ For comparison, consider a typical stablecoin cryptocurrency transaction with an average fee of about 0.30 $ and a transaction of approximately 250 bytes (B≅2000 bits). The corresponding economic dissipation per bit is ${\hat{E}}_{sc}=\frac{0.30\;\$}{2000\;\mathrm{bits}}=1.5\times 10^{-4}\frac{\$}{\mathrm{bit}}$ yielding $T_{sc}^{*}≅2.2\times 10^{-4}\frac{\$}{\mathrm{bit}}$. Thus, the traditional remittance rail is approximately five times hotter than the stablecoin rail in the Landauer sense, $\frac{T_{rem}^{*}}{T_{sc}^{*}}≅5.3$. Empirically, stablecoin usage grows precisely in corridors where remittance costs are highest, indicating that capital is re-routed from the hotter informational channel toward the colder one. This observation is consistent with the thermodynamic interpretation that economic flows are constrained by informational dissipation quantified by the Landauer-based economic temperature [42].

Consider that the economic temperature $T^{*}$ (whether marginal or actual) does not represent a physical thermodynamic temperature. Rather, it measures the minimal monetary cost associated with one irreversible bit-level operation in a given economic infrastructure. In practical electronic systems most energy dissipation arises from conventional electrical losses (resistive heating, switching losses, leakage currents), which typically exceed the Landauer limit by many orders of magnitude. The Landauer principle therefore does not describe the dominant physical dissipation mechanism but rather provides a fundamental lower bound on the energy required for irreversible information processing. We do not separate the microscopic Landauer dissipation from ordinary electrical losses (e.g., Joule heating); rather, the electricity price provides an empirical estimate of the minimal economic cost of irreversible information processing, allowing the economic temperature to be defined as the monetary cost per processed/erased bit.

Economic decisions are usually complex processes and typically involve more than a one “bit” decision. It should be emphasized, that the Landauer limit holds also for many-valued logic systems [43]. It is also important that in realistic economic networks a firm interacts with multiple suppliers and consumers through different informational channels. Each channel may therefore possess its own actual economic temperature $T_{act}^{*}$. In analogy with thermodynamics, economic flows are governed primarily by temperature differences between interacting channels, rather than by the absolute value of temperature itself.

### 2.5. Economic Szilárd Engine

**Figure 1:**
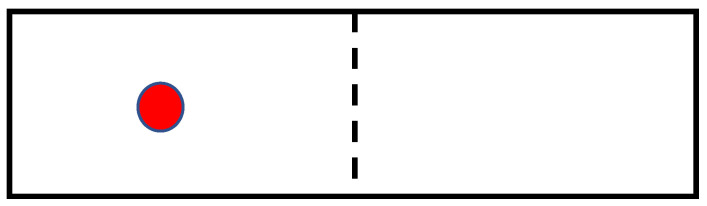
The minimal thermodynamic Szilard’s engine is depicted. The engine consists of a single molecule in a box connected to a heat bath at temperature *T*. Partition divides the box into two equal sub-boxes.

We now consider a minimal economic Szilard engine. Thermodynamic Szilard’s minimal engine is the cleanest possible thought experiment showing why the Landauer limit is unavoidable [44,45,46]. The engine consists of a single molecule in a box connected to a heat bath at temperature *T* (Figure 1).

We insert a partition, measure which side the molecule is on (that is 1 bit of information), and then let the molecule expand isothermally, pushing a piston. From this expansion we extract exactly $W=k_{B}Tln2$ of work. So far it looks like a violation of the Second Law, indeed, we extract work from a single heat bath. Landauer’s insight closes the loop: to run the engine cyclically, the measurement record (the 1 bit) must be erased. Erasing 1 bit dissipates $E_{min}=k_{B}Tln2$, as heat which is exactly the Landauer limit.

Now we introduce a minimal economic Szilárd engine. We propose to replace physical elements with economic ones, according to Table 1. Now the particle position corresponds to the market state, described by the binary alternative, the partition corresponds to the decision boundary and memory erasure corresponds to the decision finalization/record overwrite.

Now we introduce minimal economic Szilard engine. The working substance of the engine is information. An economic Szilárd engine is a minimal economic system that acquires one bit of information about an economic state, conditionally executes an action based on that bit, extracts monetary work from this asymmetry, and must eventually erase the information, incurring a minimal cost $\hat{E}=T_{act}^{*}ln2.$

The economic Szilárd engine represents the minimal microscopic realization of Landauer-based economic thermodynamics. In this engine, a single bit of economic information is acquired, conditionally exploited to extract monetary profit, and subsequently erased to complete a cycle. The extracted economic work is fundamentally bounded by the Landauer cost associated with information erasure, ensuring compliance with the Second Law. High-frequency trading strategies, arbitrage mechanisms, and cryptocurrency mining may all be interpreted as realizations of economic Szilárd engines operating at different effective economic temperatures. The efficiency of the economic Szilárd engine is $\eta_{sz}=\frac{{\hat{W}}^{*}}{\hat{E}}<1$. Thus, the monetary profit of the engine is restricted by the Landauer economic limit: ${\hat{W}}^{*}<\hat{E}=T_{act}^{*}ln2$.

### 2.6. Optimization of the Power of Economic Engines

Carnot economic engine provides the upper bound on efficiency but delivers zero economic power in the reversible limit [47]. To model a maximal profit rate, we adopt finite-time (endoreversible) thermodynamics where the internal cycle is reversible while irreversibility is shifted to finite-rate exchange with economic reservoirs. The resulting optimal-power efficiency is the Curzon–Ahlborn form is supplied by Equation (12) [47]:

(12) $$\eta_{MP}=1-\sqrt{\frac{T_{actC}^{*}}{T_{actH}^{*}}},$$

which is strictly below the economic Carnot limit, established by Equation (10). As it occurs in classical thermodynamics, maximal profit rate (Carnot rate) requires finite inefficiency, in other words, ultra-efficient markets earn slowly. HFT works as a near maximum-power regime, not as a Carnot.

### 2.7. Economic Entropy

The Landauer economic principle naturally suggests a definition of economic entropy. In physical thermodynamics, entropy is introduced through the isothermal reversible heat exchange relation dQ=TdS. Within the introduced economic framework, the analogous quantity is the minimal monetary dissipation associated with irreversible information processing. If erasure of one bit costs $\hat{E}=T^{*}ln2,$ then economically isothermal processing/erasure of *n* bits yields:

(13) $$\hat{Q}=nT^{*}ln2,$$

where $\hat{Q}$ is the minimal monetary dissipation, $\left[\hat{Q}\right]=\$$. We adopt the convention that $\hat{Q}$ represents economic heat outflow, i.e., irreversible monetary dissipation leaving the system. This motivates the definition of the economic entropy $S_{e}$ as:

(14) $$S_{e}=nln2.$$

or, in differential form:

(15) $$dS_{e}=dnln2.$$

Economic entropy defined with Equation (14) is dimensionless. It is a linear measure of the amount of lost or erased information. Equation (15) shows that each irreversibly processed bit contributes a fixed entropy increment ln2, so that economic entropy measures the cumulative number of informational decisions performed by the economic system. Thus, the minimal monetary dissipation ${\hat{Q}}_{min}$ associated with an irreversible economic information processing step satisfies:

(16) $$d{\hat{Q}}_{min}=T^{*}dS_{e}.$$

Equation (16) represents the economic analogue of the thermodynamic entropy relation, where the economic temperature $T^{*}$ measures the marginal monetary cost per processed bit. In this framework, economic entropy quantifies the cumulative amount of irreversibly erased information processed by an economic system, measured in natural units. An increase in economic entropy therefore corresponds to the cumulative irreversible processing of information required for economic decision-making, communication, and transaction validation. Equation (16) shows that any increase in economic entropy necessarily requires a positive monetary outflow.

Economic entropy defined by Equations (14) and (15) enables the re-shaping of the Second Law of Economic Thermodynamics. Consider an informationally isolated (adiabatic) system. The number of irreversibly erased bits in such a system cannot decrease. It will be constant if in the system only logically reversible processes take place. And it will increase if logically irreversible processes occur in the system. Thus, the informational content of an isolated system can either remain constant (reversible processes) or decrease due to irreversible erasure, namely Equation (17) is true:

(17) $$dS_{e}\geq 0.$$

The equality corresponds to logically reversible information processing, while the strict inequality corresponds to logically irreversible operations, such as decision overwriting, memory resetting, or transaction validation. Thus, in an informationally isolated economic system the informational content can either remain constant or decrease through irreversible erasure, which leads to an increase in economic entropy. Equation (17) establishes an economic arrow of time: in adiabatic information systems, processes evolve in time in such a way that economic entropy does not decrease.

In high-frequency trading environments, where billions of informational decisions are executed daily, the cumulative economic entropy production may therefore be interpreted as a quantitative measure of the informational irreversibility of the market.

In real economic processes the monetary dissipation generally exceeds the Landauer minimal bound because of additional irreversibilities such as latency, market impact, communication delays, and transaction costs. Therefore, for an arbitrary economic process the following Clausius-type inequality holds:

(18) $$\delta \hat{Q}\geq T^{*}dS_{e}.$$

Equation (16) describes the Landauer-limit case of logically irreversible information processing, in which the monetary dissipation attains its minimal value compatible with erasure; in general, irreversible processes satisfy the inequality (18). In real economic systems the inequality is strict, reflecting the presence of additional dissipative mechanisms such as transaction fees, spreads, infrastructure costs, and imperfect information processing. For a cyclic economic process this inequality leads to the integral form:

(19) $$\oint \frac{\delta \hat{Q}}{T^{*}}\geq 0,$$

which represents the Clausius inequality of economic thermodynamics. Here $\delta \hat{Q}$ denotes dissipated resource (outflow), hence the Clausius inequality appears with the opposite sign compared to standard thermodynamics. This result expresses the impossibility of constructing an economic engine that extracts unlimited monetary profit from informational reservoirs without producing irreversible informational dissipation.

### 2.8. First Law of Economic Thermodynamics

We already re-introduced the economic temperature, the economic entropy and economic monetary dissipation based on the Landauer principle. This is the time to define the economic internal energy $\hat{U}$. We define the economic internal energy $\hat{U}$ as the available economic resource stored in the system, for example: capital available for action, liquidity reserve, computational/infrastructural budget, stored informational advantage, exploitable economic potential, $\left[\hat{U}\right]=\$$. For the information-processing systems we adopt $\hat{U}=\hat{U}\left(T^{*}\right)$ for a single-parameter economic equilibrium description, and $\frac{1}{T^{*}}=\left(\frac{\partial S_{e}}{\partial \hat{U}}\right)$ is true.

Thus $\hat{U}$ is not just “money in an account,” but the capacity of the system to perform economically effective action. Economic heat $\delta \hat{Q}$ already introduced by Equation (18) is the irreversible monetary dissipation caused by information processing, fees, spreads, latency penalties, computational electricity cost, communication costs, validation costs, and all resource losses associated with logical irreversibility. So, this is exactly the analogue of heat: resource flow that is dissipated and not fully controllable. Let us define the economic work $\delta \hat{W}$ as an ordered, useful economic output, for example, net extracted profit, useful reallocation of capital and execution of economically useful actions, $\left[\hat{W}\right]=\$,$ $\delta \hat{R}$ is the resource input, $\left[\hat{R}\right]=\$$. So work is the part of economic resource flow that is converted into structured gain, rather than dissipated. Economic systems are fundamentally “open dissipative systems” even when informationally isolated. Thus, the first law of economic thermodynamics appears, as:

(20) $$\delta \hat{R}=\delta \hat{U}+\delta \hat{Q}+\delta \hat{W},$$

where $\delta \hat{Q}$ is given by Equation (18). Thus, the First Law of Econodynamics is formulated as follows: in economic systems implemented through physical information processing, every change in useful economic output must be balanced by changes in stored economic potential and by irreversible monetary dissipation associated with information erasure, transmission, and processing. Or alternatively: incoming economic resource $\delta \hat{R}$ is partitioned into stored potential $\delta \hat{U}$, dissipated cost $\delta \hat{Q}$ and useful output $\delta \hat{W}$. For HFT, the First Law may be read as: the system receives economic resource in the form of capital, data access, computational capability, and informational opportunities; part of this resource is dissipated irreversibly through fees, colocation, market data costs, electricity, cancellations, and latency losses; the remaining part may appear as useful economic work, namely net profit.

A real numerical example of the First Law of Econodynamics is supplied by Virtu Financial, a public high-frequency market-making firm [48]. In 2024, Virtu reported $674.426 million in brokerage, exchange, clearance and payment-for-order-flow expenses, and $236.446 million in communication and data-processing expenses, the latter including colocation, network connectivity, and market-data subscriptions. Taking these two categories as the minimal irreversible monetary dissipation, one obtains $\delta \hat{Q}=910.872\times 10^{6}\;\$$. The firm’s net income in 2024 was $\delta \hat{W}=534.535\times 10^{6}\;\$$, which we identify with useful ordered economic work. The total revenue of Virtu was reported in 2024 as approximately $\delta \hat{R}≅2.7\times 10^{9}\;\$.$

Hence the First Law, supplied by Equation (20), yields $\delta \hat{U}=\delta \hat{R}-\delta \hat{Q}-\delta \hat{W}=1.2546\times 10^{9}\;\$$. On a per-trading-day (≅250 days) basis, this corresponds to $\delta \hat{R}≅10.8\times 10^{6}\frac{\$}{\mathrm{day}}$, $\delta {\hat{Q}}_{d}≅3.64\times 10^{6}\frac{\$}{\mathrm{day}}$, $\delta {\hat{W}}_{d}=2.13\times 10^{6}\frac{\$}{\mathrm{day}},$ and $\delta {\hat{U}}_{d}=5.02\times 10^{6}\frac{\$}{\mathrm{day}}$.

The First Law of Economic Thermodynamics, given by Equation (20) enables natural definition of the economical efficiency, supplied by Equation (21):

(21) $$\eta_{ec}=\frac{\delta \hat{W}}{\delta \hat{R}}.$$

Equations (20) and (21) immediately yield:

(22) $$\eta_{ec}+\frac{\delta \hat{Q}}{\delta \hat{R}}+\frac{\delta \hat{U}}{\delta \hat{R}}=1.$$

### 2.9. Third Law of Economic Thermodynamics

In classical thermodynamics, the Third Law is usually given in one of two forms:

(i) Nernst theorem: as T→0 the entropy change associated with any isothermal reversible process tends to zero.
(ii) Unattainability principle: absolute zero cannot be reached by any finite sequence of physical processes.

In our approach economic entropy is defined as $S_{e}=nln2$, that is, by the number of irreversibly erased bits. This entropy is not equilibrium statistical entropy in the usual microscopic sense. This entropy does not depend on temperature, but it is rather a measure of irreversible information processing. Therefore, it would be incorrect to interpret this statement too literally: $\underset{T\to 0}{\lim}S_{e}\left(T\right)=const$. So, the better route is to formulate the Third Law as an unattainability principle for zero economic temperature. What is the meaning of zero economic temperature? $T^{*}=0$ would mean that irreversible processing of one bit costs nothing, information erasure or decision finalization is free, there is no monetary dissipation per economically relevant bit, one could process information without friction, one could finalize decisions without any irreversible cost. But this would contradict the internal consistency of the suggested framework, because then: costless arbitrage would become possible, costless state transitions would become possible, the Carathéodory-type inaccessibility principle would collapse, the economic Second Law would collapse, one could in principle build economic perpetual-motion schemes. So, the Third Law should say that such a state cannot be reached.

Thus, natural formulation of the Third Law of Economic Thermodynamics is shaped as follows: A state of zero economic temperature cannot be attained by any finite economic process. Or alternatively: It is impossible, by any finite sequence of economic operations, to reduce the actual economic temperature of a system to zero. This is the direct analogue of the thermodynamic unattainability principle. Since $T^{*}$ is the minimal monetary cost per irreversible bit, the statement, $T^{*}\to 0$ would mean approaching an ideal infrastructure in which information transmission is free. The suggested Third Law says that this ideal can perhaps be approached, but never exactly reached in any real finite economic system.

The physical Third Law and the Landauer principle are deeply connected. The Landauer limit gives $\hat{E}=T^{*}ln2$; $T^{*}=0$ means costless irreversible decision-making. The Third Law forbids the attainment of this state. So, the economic Third Law may be stated as: since irreversible economic information processing always requires a nonzero minimal cost, the state $T^{*}=0$ is unattainable in any realizable economic infrastructure.

In the suggested framework, “cooling” an economic system means reducing its actual economic temperature $T_{act}^{*}$, i.e., reducing the monetary cost per processed bit. This can happen through: better hardware, cheaper electricity, lower communication overhead, lower transaction fees, better protocols, more efficient market design, reduced latency, and reduced clearing/settlement costs. The Third Law says: these improvements may reduce $T_{act}^{*}$ but cannot drive it exactly to zero in finitely many steps. So, there is always some irreducible informational friction.

An economic Nernst-type statement is formulated as follows: Zero economic temperature would correspond to a market or infrastructure in which one bit of irreversible economic information could be processed at zero monetary cost. Such a state would eliminate information friction entirely and would make costless arbitrage, costless decision finalization, and dissipationless economic cycling possible. Therefore, zero economic temperature is unattainable. Thus, the Third Law expresses the fundamental impossibility of eliminating informational friction in economic systems.

## 3. Discussion

### 3.1. Constituting Information-Theoretic Paradigm of Economics with the Landauer Principle

The introduced actual economic temperature $T_{act}^{*}$ establishes a direct conceptual bridge between the Landauer economic principle and the information-theoretic paradigm of science proposed by John Archibald Wheeler [49,50]. In 1989, Wheeler formulated the paradigm known as “it from bit,” expressing the idea that physical reality emerges from information. According to this view, every physical event ultimately originates from elementary yes/no questions and their recorded answers; matter, fields, and even space-time arise as secondary constructs from informational acts. In Wheeler’s interpretation, physics is therefore not the study of things, but the study of information acquisition under physical constraints. The Landauer principle provides the quantitative backbone of this paradigm by demonstrating that information processing is inseparable from irreversibility and energy dissipation: the act of erasing or reliably transmitting a bit necessarily incurs a minimal energetic cost.

The present work extends this logic to economic systems. We propose the following reformulation: every economic effect is information-theoretic in origin. Prices, trades, arbitrage opportunities, market equilibria, and crises do not arise from material exchange alone but from information acquisition, transmission, comparison, and erasure performed by economic agents and institutions. A price change is the outcome of registered information; a trade is a decision conditioned on a finite informational input; a market equilibrium corresponds to a state where no new exploitable information is available. Within this perspective, economic activity is fundamentally an information-processing process implemented in physical hardware: data centers, communication networks, electronic exchanges, and computational devices. Consequently, economic dynamics are subject to the same fundamental limitations as physical information processing. The economic temperature $T_{act}^{*}$ defined via the Landauer-like bound $\hat{E}=T_{act}^{*}ln2$ quantifies the irreducible actual monetary cost of performing one elementary informational act in the economic system. It measures how expensive it is, in economic terms, to ask and answer a single yes–no question about the market. In this sense, $T_{act}^{*}$ plays a role analogous to physical temperature: in physics, temperature quantifies the energetic cost of microscopic disorder, in economics, $T_{act}^{*}$ quantifies the cost of microscopic information acquisition and irreversibility. High economic temperature corresponds to environments where information is expensive, noisy, delayed, or energy-intensive; low economic temperature corresponds to highly efficient informational infrastructures, though never with zero cost.

The Wheeler paradigm, thus, finds a natural economic analogue: “it from bit” → physical reality emerges from information; “economy from bit” → economic reality emerges from information. Markets do not merely exchange goods and money, they process information under irreversible constraints. The Landauer-based economic temperature formalizes and quantifies this idea and embeds it into the structure of economic thermodynamics, where irreversibility, inaccessibility, and entropy production arise not metaphorically but as direct consequences of information friction. This viewpoint positions economic thermodynamics not as an analogy borrowed from physics, but as a direct continuation of information-theoretic foundations, placing economics within the same conceptual framework that underlies modern physics.

Thus, we summarize our approach in the economic “*it from bit*” principle, formulated as follows: Every economic phenomenon originates from information-theoretic processes. More precisely, any economic effect—such as a price change, transaction, arbitrage opportunity, or market equilibrium—arises from the acquisition, transmission, processing, and irreversible erasure of information performed by economic agents and institutions.

These informational acts are implemented in physical systems and therefore obey the Landauer-type bound. Consequently, each elementary economic decision requires a nonzero minimal expenditure of resources, quantified by the economic temperature $T^{*}$. In this framework, economic systems are understood not as purely abstract exchanges of value, but as physical information-processing systems. The economic temperature $T^{*}$ measures the minimal irreversible cost of asking and answering a single yes–no economic question. Thus, economic irreversibility, transaction costs, and market frictions emerge as fundamental consequences of information theory rather than contingent institutional imperfections. Examples of the “hot” and “cold” economic markets are supplied in Appendix B and Appendix C.

We also clearly recognize the limitation of the introduced “economic temperature”. The proposed Landauer-based economic temperature $T^{*}$ has several inherent limitations. It is not a universal descriptor for all economic activity and is most meaningful only for systems in which decisions are explicitly implemented as information storage, transmission, or erasure with measurable costs. The identification of a “bit” is model-dependent and depends on the chosen level of abstraction, while the definition itself targets a minimal irreducible cost and may be far below actual economic costs dominated by institutional, technological, or regulatory overheads. Moreover, separating informational dissipation from other economic frictions (fees, spreads, risk premia) is not always unambiguous. The economic temperature is highly sensitive to infrastructure, energy prices, and market design, and may vary in time, so it should be interpreted as an effective, system- and scale-dependent quantity rather than a universal state variable. It is also possible that the universal definition of the economic temperature, applicable for all kinds of markets does not exist.

One more limitation of the introduced concept should be clearly understood. The thermodynamic cost established by the Landauer bound applies specifically to logically irreversible operations, such as erasure or overwriting of information, in which multiple possible memory states are compressed into a single state. In physical terms, this operation reduces the entropy of the information-bearing system and therefore requires a compensating entropy increase in the environment, leading to a minimal dissipation of $k_{B}Tln2$ per erased bit. In economic information-processing systems, the analogue of erasure is decision finalization, when alternative possibilities (e.g., buy/sell, route/suppress, accept/reject) are discarded and the system commits to a single realized action. By contrast, information acquisition or generation, such as observing market signals, receiving price updates, or copying data, can in principle be implemented as logically reversible operations, which only create correlations between the system and the memory without destroying information. Such operations do not require a fundamental thermodynamic cost. In real economic infrastructures these processes still consume energy and resources due to technological inefficiencies, but this cost is not imposed by the Landauer limit itself. The Landauer bound becomes unavoidable only when the system must reset its memory or overwrite previous records in order to continue operating cyclically, which corresponds to the irreversible stage of economic decision-making. Introduced economic temperature enables re-formulation of the entire set of thermodynamic laws, adopted to economic systems.

### 3.2. Guidelines for the Future Investigations

The introduced scheme of econophysics is a very preliminary one. In future investigations we plan to introduce the economic temperature $T^{*}$ for payment rails (card networks, bank transfers).

We also plan to formalize how value/“heat” flow depends not only on $T^{*}$ gradients but also on coupling strength (information bandwidth, latency sensitivity, access rights). The goal is an analogue of radiative transfer:

(23) $${\dot{Q}}^{*}\sim \int H\left(\nu \right)\left(T_{H}^{*}\left(\nu \right)-T_{C}^{*}\left(\nu \right)\right)d\nu ,$$

where ${\dot{Q}}^{*}$ is a value/heat flux (profit-rate analogue), H(ν) captures market microstructure coupling and ν is an “information frequency” scale (decision cadence).

It is also desirable to move beyond equilibrium-like statements toward: (i) entropy production rates from fees/spreads/slippage, (ii) fluctuation relations for profit and loss (P&L) distributions, (iii) establishment finite-time bounds linking profit rate and dissipation rate (a “thermodynamic uncertainty relation” style program for markets).

## 4. Conclusions

In this work, we introduced a Landauer-based definition of economic temperature and demonstrated that it provides a physically grounded and operational foundation for extending thermodynamic reasoning to economic systems. Unlike traditional economic notions of temperature—based on volatility, rationality, entropy, or utility—our definition does not rely on behavioral assumptions, equilibrium hypotheses, or subjective agent models. Instead, it rests on a universal structural feature shared by physical and economic systems: information processing is logically irreversible and incurs an irreducible cost. By reformulating the Landauer principle in economic terms, we defined the economic temperature $T^{*}$ through the minimal monetary cost associated with erasing or transmitting one bit of information. This definition ties economic temperature directly to measurable physical quantities such as electricity prices, computational infrastructure, and communication efficiency. As a result, $T^{*}$ emerges as a technological and infrastructural parameter, rather than a psychological or institutional one. Any computation, whether conventional or unconventional, consumes energy [51,52]; thus,$T^{*}$ may be introduced for a broad range of computation based economic systems. Marginal and actual economic temperatures are distinguished. Actual minimal cost of erasure on 1 bit of information, in a given economic system, may be much larger than the marginal cost, defined by the cost of electrical power, required for erasure of 1 bit.

Using this definition, we reinterpreted the major formulations of the Second Law of Thermodynamics in an economic context. The Clausius formulation was shown to correspond to the impossibility of spontaneous value flow from colder to hotter economic subsystems without dissipation. The Carathéodory formulation naturally translated into an accessibility principle: every equilibrium economic state is surrounded by nearby states that cannot be reached without spending money or information. This inaccessibility arises from the Landauer cost of information acquisition and decision-making, even for infinitesimal economic changes.

We further constructed economic analogues of the Carnot engine, Szilárd engine, and finite-time heat engines. These models demonstrate that profit extraction in markets is fundamentally constrained by information thermodynamics, leading to strict upper bounds on efficiency and power. In particular, strategies such as high-frequency trading and cryptocurrency mining can be viewed as economic information engines, converting information erasure into monetary profit while necessarily producing economic “heat” in the form of transaction costs, fees, latency losses, and energy dissipation. Economic perpetual motion machines of the second kind—strategies promising unlimited profit without dissipation—are therefore forbidden.

Finally, we connected the proposed framework to the information-theoretic paradigm of science, reformulating the “it from bit” principle for economics. In this view, economic reality emerges from information: prices, trades, arbitrage opportunities, and equilibria are outcomes of information acquisition, processing, and erasure implemented in physical systems. The economic temperature $T^{*}$ quantifies the minimal irreversible cost of these informational acts and thus plays a role analogous to physical temperature in thermodynamics.

Physically, the minimal Landauer cost applies to logically irreversible operations such as information erasure, whereas reliable transmission of a bit through a real channel usually requires additional energy associated with signal generation, noise suppression, and error correction. The same distinction appears in economic systems: decision finalization represents the analogue of information erasure, while market data transmission, order routing, or ledger updates correspond to information transport and typically incur much larger infrastructure-dependent costs. Consequently, the empirically observed economic temperature reflects an effective channel cost that generally lies far above the Landauer lower bound.

Economic entropy is introduced via the Landauer principle and measures the cumulative amount of information irreversibly erased within an economic system. This definition naturally leads to the concept of an economic arrow of time: in informationally isolated (adiabatic) economic systems, processes evolve in time in such a way that economic entropy does not decrease.

Introducing economic entropy enabled formulation of the First and Third Laws of economic thermodynamics. Zero economic temperature would correspond to a market or infrastructure in which one bit of irreversible economic information could be processed at zero monetary cost. Such a state would eliminate information friction entirely and would make costless arbitrage, costless decision finalization, and dissipationless economic cycling possible. Zero economic temperature is unattainable.

In summary, the Landauer-based economic temperature provides a unifying, physically grounded framework for econophysics. It explains economic irreversibility, transaction costs, and market frictions as fundamental consequences of information theory rather than contingent imperfections. This approach opens a path toward a genuinely microscopic foundation of economic thermodynamics and places economic systems within the same conceptual structure that underlies modern physics. The limitations of the introduced economic temperature are addressed.

## Figures and Tables

**Table 1:** Analogy between physical Szilárd engine and its economic analogue.

Physical Szilárd engine	Economic analogue
Particle position	Market state (binary alternative)
Partition	Decision boundary
Measurement	Information acquisition
Memory erasure	Decision finalization/record overwrite
Heat bath	$\mathrm{Economic}\;\mathrm{reservoir}\;\mathrm{at}\;T_{act}^{*}$

## Data Availability

The original contributions presented in this study are included in the article. Further inquiries can be directed to the corresponding author.

## Abbreviations

The following abbreviations are used in this manuscript:

BTC	Bitcoin
BTC price	Market price of one bitcoin unit.
HFT	High-frequency trading
vB	Virtual byte
PHLX	Philadelphia Stock Exchange
PSX	Nasdaq PSX (Nasdaq PSX Exchange); an equity trading venue operated by Nasdaq

## Appendix A. Calculation of Economic Temperature

We already calculated the marginal economic temperature with Equation (5) as $T_{ind}^{*}=9.69\times 10^{-29}\frac{\$}{\mathrm{bit}}$ for the industrial price and $T_{com}^{*}=2.52\times 10^{-28}\frac{\$}{\mathrm{bit}}$ for the commercial price of electricity. Typical system-level energy per bit often quoted around $10^{-14}\frac{J}{\mathrm{bit}}$, with an aspirational lower bound around $10^{-17}\frac{J}{\mathrm{bit}}$ [49]. Table A1 summarizes economic temperatures for the ideal and real/actual economic systems.

**Table A1:** Economic temperatures calculated for the ideal and real economic systems.

Electricity Price	Economic System Regime	Economic Temperature, $T^{*},\frac{\$}{\mathrm{bit}}$
Industrial	Landauer/ideal	$9.69\times 10^{-29}\frac{\$}{\mathrm{bit}}$
Commercial	Landauer/ideal	$2.52\times 10^{-28}\frac{\$}{\mathrm{bit}}$
Industrial	Typical real system level	$3.38\times 10^{-22}\frac{\$}{\mathrm{bit}}$
Commercial	Typical real system level	$5.29\times 10^{-22}\frac{\$}{\mathrm{bit}}$
Industrial	Aspirational level	$3.38\times 10^{-25}\frac{\$}{\mathrm{bit}}$
Commercial	Aspirational level	$5.29\times 10^{-25}\frac{\$}{\mathrm{bit}}$

## Appendix B. Calculation of Economic Temperature for Bitcoin-like Systems

One way to analyze a Bitcoin-like (on-chain) system is to treat the “information channel” as blockspace, where the marginal cost to write/transmit data is the transaction fee per virtual byte (vB), i.e., sats/vB. Let the fee rate be *f* sats/vB and BTC price (for 8 March 2026) be $P_{BTC}$, $\left[P_{BTC}\right]=\frac{\$}{BTC};1\;\mathrm{BTC}=10^{8}\;\mathrm{sats}.$ Then cost per vB: $\frac{\$}{vB}=f\times \frac{P_{BTC}}{10^{8}}$. Cost per bit (since 1 vB ≅1 byte = 8 bits), ${\hat{E}}_{BTC}\left[\frac{\$}{\mathrm{bit}}\right]=\frac{1}{8}f\times \frac{P_{BTC}}{10^{8}}$. Hence economic temperature is given by: $T_{BTC}^{*}=\frac{{\hat{E}}_{BTC}}{ln2}=\frac{1}{8ln2}f\frac{P_{BTC}}{10^{8}}.$ Using a current BTC price $P_{BTC}≅65,053\frac{\$}{BTC}$, and averaged transaction fee ≅ 2.5 sats/vB and median ≅ 0.2 sats/vB, supplied by BitInfoCharts currently reports, we calculate for the median conditions ($f=0.2\frac{sats}{vB}$) $T_{BTCM}^{*}≅2.35\times 10^{-5}\frac{\$}{\mathrm{bit}}$, and for the average conditions ($f=2.5\frac{sats}{vB}$) $T_{BTCA}^{*}≅2.93\times 10^{-4}\frac{\$}{\mathrm{bit}}$. These $T_{act}^{*}$ values are enormous compared to the marginal ones calculated in Appendix A electricity/Landauer-limit example (order $10^{-28}\frac{\$}{\mathrm{bit}}$), or actual/real computing systems (order $10^{-22}\frac{\$}{\mathrm{bit}}$). This means that Bitcoin-like settlement is an extremely “hot” economic channel (high marginal cost per bit committed to the ledger).

## Appendix C. Economic Temperature of the Mobile Internet Data Market in Israel

A commonly cited estimate puts the average price of 1 GB of mobile data in Israel at about $0.02/GB. So the monetary cost per bit is ${\hat{E}}_{MI}=\frac{0.02}{8\times 10^{9}}\frac{\$}{\mathrm{bit}}=2.5\times 10^{-12}\frac{\$}{\mathrm{bit}}$. Thus, the actual economic temperature equals: $T_{MI}^{*}=\frac{{\hat{E}}_{MI}}{ln2}≅3.6\times 10^{-12}\frac{\$}{\mathrm{bit}}$. This economic temperature is much smaller than that calculated for Bitcoin-like systems, estimated in Appendix B. Thus, we conclude that the mobile internet data market is much more “cold” than the bitcoin market.
